# The ALS-Related σ1R E102Q Mutant Eludes Ligand Control and Exhibits Anomalous Response to Calcium

**DOI:** 10.3390/ijms21197339

**Published:** 2020-10-04

**Authors:** María Rodríguez-Muñoz, Elsa Cortés-Montero, Javier Garzón-Niño, Pilar Sánchez-Blázquez

**Affiliations:** Neuropharmacology, Cajal Institute, CSIC, Avenida Doctor Arce, 37. 28002 Madrid, Spain; mrodriguez@cajal.csic.es (M.R.-M.); elsa.cortes@cajal.csic.es (E.C.-M.); jgarzon@cajal.csic.es (J.G.-N.)

**Keywords:** sigma type receptor 1, juvenile amyotrophic lateral sclerosis, E102Q mutation, *N*-methyl-D-aspartate receptor, transient receptor potential calcium channels, binding immunoglobulin protein

## Abstract

Sigma receptor type 1 (σ1R) is a transmembrane protein expressed throughout the central nervous system and in certain peripheral tissues. The human σ1R E102Q mutation causes juvenile amyotrophic lateral sclerosis (ALS), likely by inducing a series of alterations in calcium efflux from the endoplasmic reticulum (ER) to mitochondria that affects calcium homeostasis and cellular survival. Here, we report the influence of calcium on σ1R E102Q associations with glutamate *N*-methyl-D-aspartate receptors (NMDARs), binding immunoglobulin protein (BiP), and transient receptor potential calcium channels A1, V1, and M8. The mutant protein inhibited the binding of calmodulin to these calcium channels and interacted less with BiP than wild-type σ1R, thereby contributing to calcium homeostasis dysfunction. Mutant σ1R, but not wild-type σ1R, strongly bound to histidine triad nucleotide binding protein 1, which regulates neuromuscular synaptic organization and target selection through teneurin 1. While ligands regulated the association of σ1R wild-type with NMDARs and BiP, they failed to modulate the interaction between these proteins and the σ1R E102Q mutant. Thus, the σ1R E102Q mutant exhibited an anomalous response to cytosolic calcium levels, altered affinity for target proteins, and a loss of response to regulatory ligands. We believe that these modifications may contribute to the onset of juvenile ALS.

## 1. Introduction

The sigma receptor type 1 (σ1R) is a 223-amino-acid polypeptide that is widely distributed in both the central and peripheral nervous systems [1,2,3,4]. This protein is encoded by the *SIGMAR1* gene and was initially described as an opioid receptor on the plasma membrane of neurons [5,6,7]. Later, σ1R was also found in the endoplasmic reticulum (ER) [8] and the nuclear envelope [9,10]. σ1R participates in several processes, such as neuronal survival, ion channel activity, Ca^2+^ signaling, synaptic plasticity, memory, and drug addiction [11,12,13]. Furthermore, it has also been implicated in central nervous system pathologies, including amnesia [13], pain [14], depression [15], schizophrenia [16], stroke [17], retinal neuron degeneration [18,19], and Alzheimer′s [20], Parkinson′s [21], and Huntington′s [22] diseases.

Different disorders are associated with known mutations in human *SIGMAR1*, including frontotemporal lobar degeneration (FTLD) [23] and motor neuron diseases such as autosomal recessive distal hereditary motor neuropathy [24] and juvenile amyotrophic lateral sclerosis (ALS) [25,26]. σ1R is enriched in motor neurons in the brainstem and spinal cord [27]. Accordingly, σ1R knockout mice exhibit deficits in motor control caused by motor neuron degeneration in the spinal cord [28], and loss of σ1R exacerbates ALS progression in G93A-SOD1 mice [26]. σ1R establishes Ca^2+^-dependent associations with a series of signaling proteins, such as binding immunoglobulin protein (BiP), at mitochondria-associated ER membranes (MAMs). Upon ER Ca^2+^ depletion or ligand stimulation, σ1R dissociates from BiP, leading to prolonged Ca^2+^ influx into mitochondria via inositol 1,4,5-triphosphate receptor type 3 (IP3R3) [11]. A single missense mutation in the second exon of the *SIGMAR1* gene is associated with the juvenile form of ALS [25]. This mutation, which involves the substitution of glutamine for glutamic acid at position 102 (E102Q) of σ1R, is located in the linker region between β2 and β3 [29]. A series of recent studies have shed some light on the molecular mechanisms by which the σ1R E102Q mutation causes ALS. In vitro, the σ1R E102Q mutant is prone to aggregate, and in transfected cells, the mutant is unstable and incapable of binding to IP3R3 in the ER membrane [26]. Moreover, σ1R E102Q accumulates in the ER and associated compartments, provoking alterations in proteasomal degradation and calcium homeostasis [30].

Different classes of calcium channels in the plasma membrane dynamically control intracellular calcium levels, such as tetrameric glutamate *N*-methyl-D-aspartate receptors (NMDARs) and transient receptor potential (TRP) family channels. Notably, σ1R binds to the NR1 subunit of inotropic glutamate NMDARs [31,32] and, together with histidine triad nucleotide-binding protein 1 (HINT1), coordinates the activity of G-protein coupled receptors (GPCRs), such as the mu-opioid receptor (MOR), with that of NMDARs [32]. A similar mechanism also applies to neural TRP ankyrin member 1 (TRPA1), TRP vanilloid member 1 (TRPV1), and TRP melastatin member 8 (TRPM8). Recent data showed that σ1R and Calmodulin (CaM) bound directly to cytosolic regions of these TRP channels [33]. The binding of σ1R to proteins such as the C1 cytosolic region of NMDAR’s NR1 subunit (NR1-C1) [31,32], TRP channels [33], and the protein BiP in the ER [11] is regulated by calcium. An increase in the calcium level promotes the interaction between σ1R and these third partner signaling proteins, while calcium depletion hinders these associations.

Specifically, σ1R competes with Ca^2+^-activated CaM and HINT1 to bind to the cytosolic C0-C1-C2 region of the NR1 subunit [31] and TRP channels [33]. As a result, σ1R promotes the activity of these calcium channels, while HINT1 and particularly Ca^2+^-CaM diminish their activity. While there is no cure for ALS, its progression can be delayed by drugs such as riluzole, which hinders the function of NMDARs [34]. It is possible that the σ1R E102Q mutant exacerbates NMDAR activity in motor neurons, where σ1R is particularly abundant [27].

These observations prompted us to study whether human σ1R E102Q mutant establishes dysregulated interactions with plasma membrane proteins that influence cytosolic Ca^2+^ levels. Thus, BiP, the NR1-C1 subunit of NMDARs, the MOR, the HINT1 protein, and the N- and C-terminal cytosolic regions of TRPA1, TRPV1, and TRPM8 channels were selected for in vitro assays. Whether the interactions between the σ1R E102Q mutant and various signaling proteins are regulated by calcium levels and the capacity of σ1R ligands to modify the interaction between the σ1R mutant and the NR1 subunit or BiP were also investigated. Compared to wild-type (WT) σ1R, the E102Q mutant receptor exhibited a stronger association with its partner proteins, except for BiP and TRPV1 C terminus (Ct), which interacted with the E102Q mutant at a much lower level than with WT σ1R. Moreover, the interactions between σ1R E102Q and its partner proteins were much less dependent on Ca^2+^ than those between WT σ1R and the same proteins, and the σ1R ligands studied did not disrupt the association of σ1R E102Q with NR1 subunits or BiP protein.

## 2. Results

**Figure 1:**
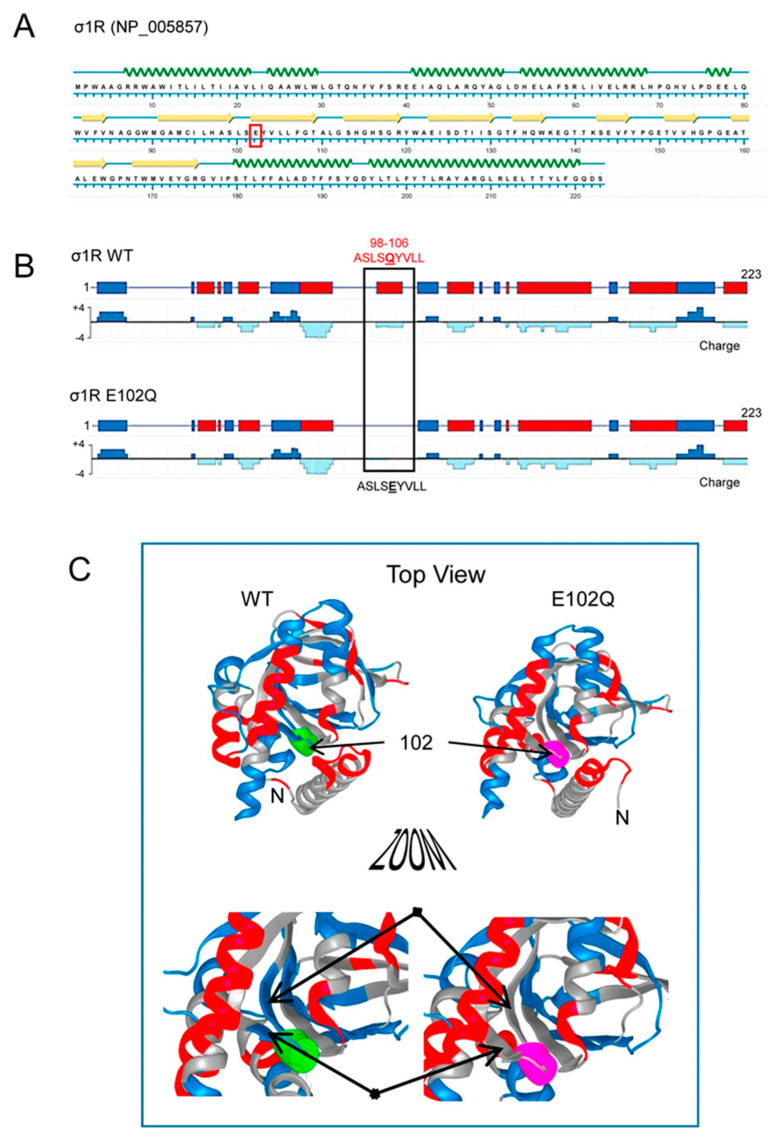
Secondary structure, charge distribution map, and 3D model of sigma receptor type 1 (σ1R). (**A**) Protein sequence and secondary structure of σ1R, with helices indicated by green waves and β-sheets by yellow arrows. The amino acid change in human σ1R associated with the juvenile form of amyotrophic lateral sclerosis (ALS) is indicated in red. (**B**) Charge distribution map of wild-type (WT) σ1R and σ1R E102Q (the images were created using NovaFold v17, DNASTAR, Inc., Madison, WI, USA). Positive charges are indicated by blue and negative charges are indicated by red. The E102Q mutant protein lacks a negatively charged cluster located in the linker region between β2 and β3 (residues 98–106, ASLSEYVLL). (**C**) 3D structure of σ1R showing the WT and mutated amino acid as a colored tube. E102 is indicated by green and Q by pink. The structural model of σ1R and its secondary structure shown here were generated with NovaFold v. 17 (DNASTAR).

Human σ1R is composed of 223 amino acids that form seven helices and ten β-sheets, with the rest of the protein being linear sequences (Figure 1A; NovaFold v17, DNASTAR). The σ1R E102Q mutant lacks a negatively charged cluster located in the linker region between β2 and β3 (residues 98–106, ASLSEYVLL), which may be essential for electrostatic interactions with partner proteins (Figure 1B,C).

**Figure 2:**
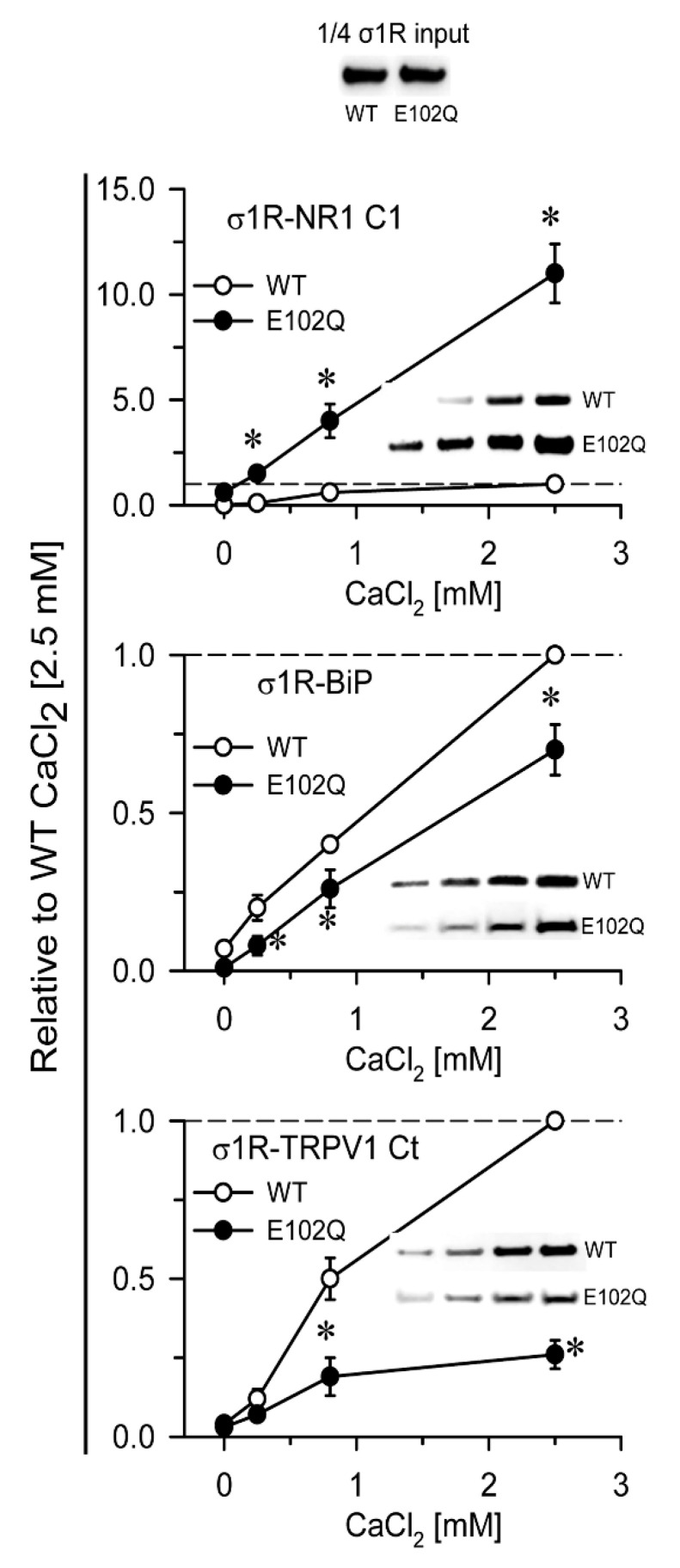
Calcium-dependent binding of σ1R WT and σ1R E102Q to the NMDAR NR1 subunit, BiP, and TRPV1 Ct. Recombinant C0-C1-C2 of the NR1 subunit, BiP, and TRPV1 Ct were covalently attached to N-Hydroxysuccinimidy (NHS)-activated Sepharose® and incubated with 100 nM WT σ1R or its mutant form in the presence of increasing amounts of calcium chloride (0, 0.25, 0.83, and 2.5 mM). The prey proteins alone (negative controls) did not bind to NHS-activated Sepharose. The pellets obtained were processed to measure σ1R expression by western blotting (see Section 4 and Figure S1). The assays were performed at least twice, and each sample was analyzed in duplicate. The association of WT σ1R or σ1R E102Q with the covalently attached proteins (NR1, BiP, and TRPV1) at different concentrations of calcium is shown relative to the association of WT σ1R with the proteins in the presence of 2.5 mM calcium (assigned an arbitrary value of 1). * indicates significant differences compared to the WT σ1R group and the corresponding concentration of calcium; all data were analyzed by ANOVA followed by the pairwise Holm–Sidak multiple comparison test; *p* < 0.05; σ1R, sigma receptor type 1; NMDAR, *N*-methyl-D-aspartate receptor; NR1-C1, cytosolic region of the C0-C1-C2 domains of the NR1 subunit of NMDARs; BiP, binding immunoglobulin protein; TRPV1 Ct, cytosolic C-terminal domain of transient receptor potential calcium channel type V1; WT, wild-type.

In in vitro assays, σ1R interacts with a series of signaling proteins, such as the cytosolic C0-C1-C2 region of the NR1 subunit of glutamate [31,32], BiP [11], the C-terminal sequence of MOR [32], and the N- and C-terminal cytosolic domains of TRPA1, TRPM8, and TRPV1 channels [33] in a calcium-dependent manner. Increases in calcium levels promote the interaction of σ1R with third partner signaling proteins, while calcium depletion inhibits these associations. The human E102Q mutant also exhibited calcium-dependent associations with the NR1-C1 subunit, BiP, and the C-terminal sequence of TRPV1. σ1R E102Q and WT σ1R differed in their binding affinity for these proteins. The mutant had a much higher affinity than the WT for the NR1-C1 subunit, while the opposite was observed for BiP and TRPV1 Ct (Figure 2).

**Figure 3:**
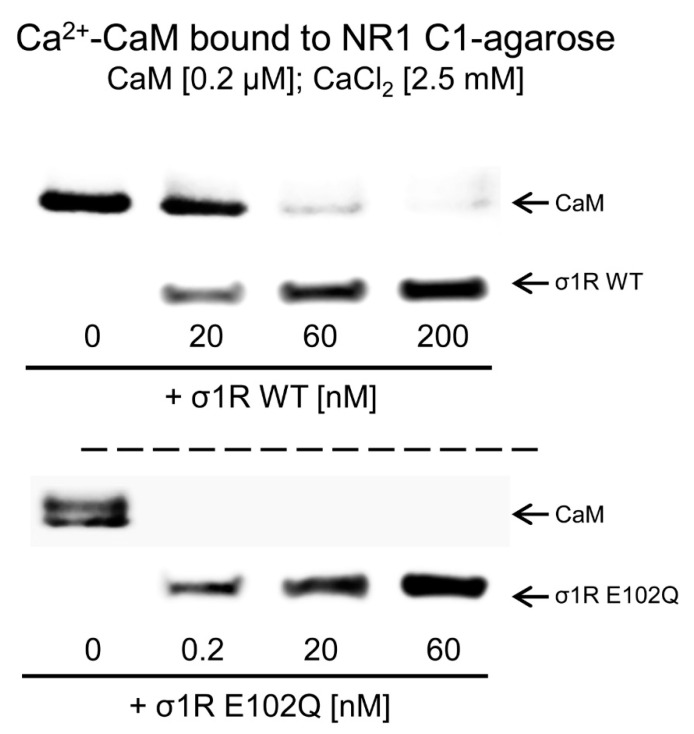
Competition analysis of σ1R and CaM for binding to the NR1-C1 subunit of NMDARs. CaM (0.2 μM) was incubated with the NR1-C1 subunit, which was covalently attached to NHS-activated Sepharose® in the presence of 2.5 mM CaCl_2_ and increasing concentrations of WT σ1R or the human σ1R E102Q mutant. After incubation, CaM and σ1R that remained bound to the NR1-C1 subunit were isolated, resolved by SDS-PAGE and analyzed by western blotting. The assays were repeated at least twice, and comparable results were obtained. Representative blots are shown. CaM, calmodulin; σ1R, sigma receptor type 1; WT, wild-type.

The activation of CaM by calcium rapidly regulates different signaling pathways and the activities of various proteins, such as NMDARs and TRP channels. The CaM-binding motifs in the NMDAR NR1-C1 subunit and TRPA1, TRPV1, and TRPM8 overlap with the binding sites of σ1R, and thus, σ1R competes with CaM for binding to specific sequences in the cytosolic regions of these proteins [31,33]. We observed that, in the presence of 2.5 mM CaCl_2_, increasing concentrations of WT σ1R and the E102Q mutant reduced the binding of calcium-activated CaM to the cytosolic C0-C1-C2 region of the NR1 subunit. While binding of 200 nM CaM was abolished by 60 nM WT σ1R, the E102Q mutant, at a concentration less than 0.2 nM, prevented CaM binding to the NR1 protein (Figure 3).

**Figure 4:**
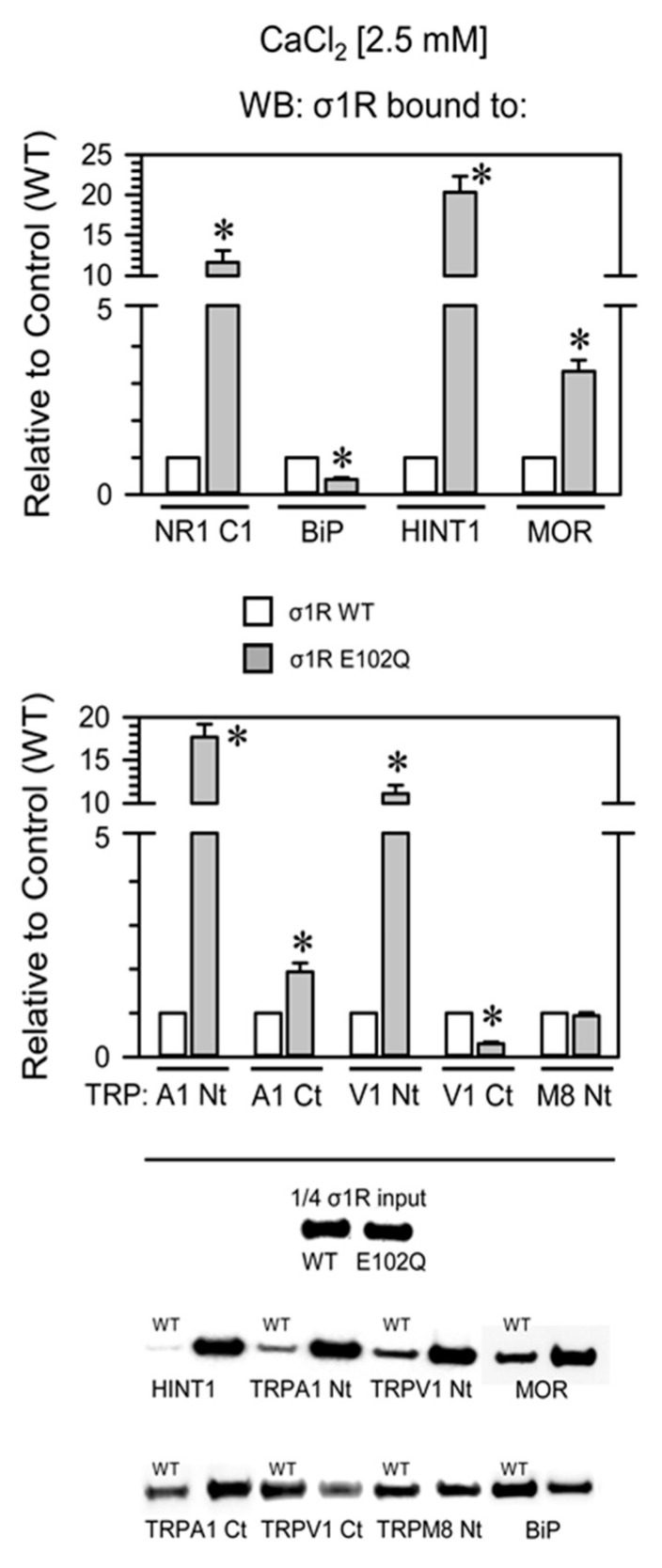
Binding of WT σ1R and σ1R E102Q to proteins that regulate NMDAR function and to the cytosolic domains of TRPA1, TRPV1, and TRPM8. Recombinant cytosolic C0-C1-C2 region of the NMDAR NR1 subunit, the C-terminal cytosolic region of MOR, the HINT1 protein, BiP, and the N- and C-terminal cytosolic domains of TRPA1, TRPV1, and TRPM8 channels were covalently attached to agarose and incubated with human WT σ1R or its mutant in the presence of 2.5 mM CaCl_2_ (details as in Figure 2). The assays were repeated at least twice, and comparable results were obtained. Representative blots are shown. For each interaction between σ1R WT or the σ1R E102Q mutant and a given protein, * indicates a significant difference compared to the σ1R WT group (assigned an arbitrary value of 1); all data were analyzed by ANOVA followed by the pairwise Holm–Sidak multiple comparison test; *p* < 0.05; σ1R, sigma receptor type 1; NR1-C1, cytosolic region the C0-C1-C2 domains of the NR1 subunit of NMDARs; BiP, binding immunoglobulin protein; HINT1, histidine triad nucleotide binding protein 1; MOR, mu-opioid receptor; TRP, cytosolic transient receptor potential types A1, V1, and M8; Ct, C-terminal domain; Nt, cytosolic N-terminal domain; WT, wild-type.

Compared with WT σ1R, the E102Q mutant bound more strongly to the NR1-C1 subunit, the HINT1 protein, and the N-terminal domains of TRPA1 and TRPV1 and with lower affinity to the C-terminal sequences of MOR and TRPA1. The opposite was observed for BiP and the C-terminus of TRPV1, and both forms of σ1R displayed similar binding to the N-terminus of TRPM8 (Figure 4).

**Figure 5:**
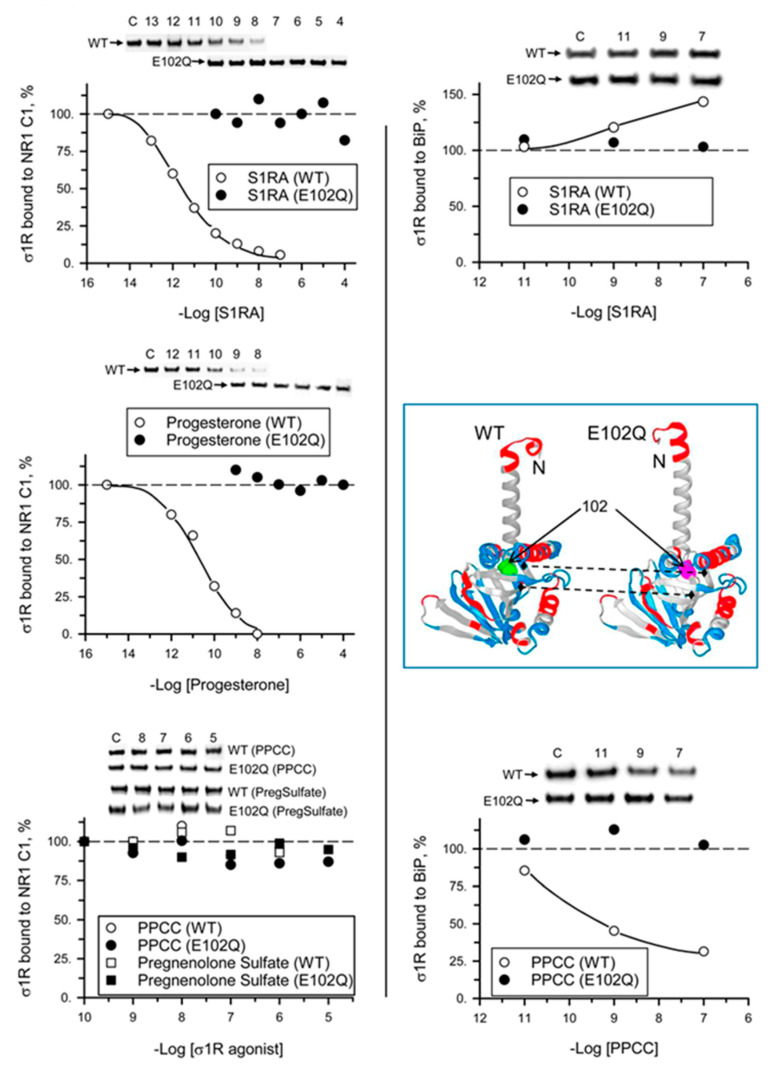
Effect of σ1R ligands on the interactions of WT σ1R and the E102Q mutant with the NR1-C1 subunit and BiP. Recombinant C0-C1-C2 region of NR1 and BiP were covalently attached to NHS-activated Sepharose® and incubated with WT σ1R or mutant σ1R (100 nM). Unbound σ1R was removed by three cycles of washing/resuspension. The protein–σ1R complexes were incubated for 30 min with rotation at room temperature (RT) in the presence of increasing concentrations of σ1R ligands in a final volume of 300 μL (50 mM Tris-HCl, pH 7.5, and 0.2% 3-[(3-cholamidopropyl)dimethylammonio]-1-propanesulfonate (CHAPS)), and 2.5 mM CaCl_2_ was used throughout the procedure. Finally, σ1R that remained attached to the NR1 subunit or BiP was resolved by SDS-PAGE and evaluated by immunoblotting. The assays were performed two times, and the samples were analyzed in duplicate for each ligand concentration. Representative blots are shown. Inset, WT σ1R and the E102Q mutant showing position 102 (solid arrows) and the loss of a negative charge in the mutant surrounding the Q residue (dashed arrows) (Novafold v. 17; DNASTAR Inc., Madison, WI, USA).

We analyzed the ability of σ1R ligands to regulate the interactions of mutant σ1R with the cytosolic C0-C1-C2 region of the NMDAR NR1 subunit and with BiP in the presence of 2.5 mM CaCl_2_. While σ1R antagonists such as progesterone and S1RA greatly reduced the σ1R WT-NR1 subunit interaction in a concentration-dependent manner [35], they did not have an effect on the σ1R E102Q-NR1 subunit interaction. The agonists PPCC and pregnenolone sulfate did not significantly alter the interaction between WT σ1R or E102Q and the NR1 subunit. On the other hand, S1RA augmented and PPCC reduced the association of WT σ1R with BiP; however, these ligands failed to alter the σ1R E102Q–BiP interaction (Figure 5).

## 3. Discussion

σ1R physically interacts with a series of cytosolic signaling proteins associated with the ER and mitochondrial calcium regulation, such as ankyrin B, IP3R3, and BiP [11,36]. At the mitochondria-associated ER membrane, σ1R associates with BiP, and depletion of ER calcium or ligand binding to σ1R disrupts this association, leading to prolonged calcium-mediated cellular signaling in mitochondria via IP3R3 [11]. Thus, σ1R deficiency results in deregulation of calcium homeostasis at the ER–mitochondria interface through mislocalization of IP3R3. Notably, in N2a-IP3R3 cells, σ1R E102Q does not interact with IP3R3, and upon ATP stimulation, the regulatory effect of the ALS-linked mutant on calcium release into the cytoplasm and mitochondria upon ATP stimulation is completely abolished [26].

In our study, the σ1R E102Q mutant showed an anomalous dependence of calcium levels to establish associations with target proteins. Thus, at low calcium levels, WT σ1R showed little or no association with the NR1 subunit, but the E102Q mutant strongly bound to this protein. Differences in competition with calcium-activated CaM for binding to the NR1 subunit were also observed between WT σ1R and the mutant. These observations indicated that σ1R E102Q prevailed over the NMDAR inhibitor Ca^2+^-CaM in binding to the NR1 subunit [37], thus increasing the entrance of calcium toward the cytosol. σ1R in the ER membrane binds BiP in a calcium-dependent manner, and this binding is retained through the Ca^2+^-regulated C-terminal domain (residues 112–223) of σ1R [11]. Our study indicated that σ1R E102Q had lower affinity for BiP than WT σ1R, which is consistent with the in vivo finding that σ1R E102Q poorly colocalizes with the BiP protein [38]. Thus, σ1R E102Q mutation may also increase calcium flux into mitochondria, contributing to the deregulation of intracellular calcium homeostasis.

It has been described that the binding of the σ1R to TRPs may favor the open probability of the channel, while CaM will reduce TRP activity by competing and diminishing σ1R binding. Hence, the resulting activity of the TRP calcium channels may depend on the concentrations of CaM and σ1R in their cytosolic environment [33]. We found that the influence of calcium on the formation of the σ1R E102Q–TRPV1 Ct complex was different than that on the formation of the WT σ1R-TRPV1 Ct complex; however, the influence that such a modification has on intracellular calcium signaling requires further research. Similarly, the σ1R mutant may block the ability of Ca^2+^-CaM to access the regulatory N-terminal cytosolic sequences of TRPA1 and TRPV1, through which CaM mainly reduces the influx of extracellular calcium [33]. However, σ1R promotes the binding of CaM to TRPA1 Ct [33], and σ1R E102Q may moderately enhance the binding of Ca^2+^-CaM to this cytosolic region. On the other hand, the mutant increased its association with TRPV1 N terminus (Nt), but reduced its binding to the C-terminal sequence of TRPV1. Overall, σ1R E102Q deregulated the binding of Ca^2+^-CaM to TRPA1 and TRPV1 channels.

On the other hand, the affinity of the σ1R E102Q mutant for MOR and especially the HINT1 protein was higher than that of WT σ1R. A series of GPCRs, such as the MOR, regulate NMDARs, inducing glutamate neural activity [35]. Thus, the HINT1–σ1R protein complex links the activity of MOR with that of NMDARs [31,32]. σ1R impairs the ability of Ca^2+^-CaM to reduce the opening probability of NMDARs and consequently to inhibit calcium influx [37]. Therefore, σ1R promotes NMDAR activity, while Ca^2+^-CaM and HINT1 diminish NMDAR activity. The human σ1R E102Q mutant may deregulate the interactions of NMDARs with other signaling proteins, contributing to neurological disorders, including neurodegenerative diseases [39], and likely to alterations in motor coordination. The progression of ALS can be delayed by drugs that inhibit the function of NMDARs [34]. Interestingly, there was a strong association between HINT1 and the σ1R E102Q mutant. The WT σ1R barely forms stable associations with HINT1 [31,32], and thus, the mutant may alter the physiologically stable associations of HINT1 with other signaling proteins. HINT1 is a zinc- and CaM-regulated SUMO protease [40], and a series of HINT1 mutants have been reported to cause human autosomal recessive axonal neuropathy with neuromyotonia [41]. The association of the σ1R mutant with the HINT1 protein is relevant to the normal functioning of motor pathways. HINT1 couples with the intracellular domain of teneurin 1, which acts as a transcription factor in the nucleus [42]. The teneurin family (four members in humans, Ten1–4) promotes neurite outgrowth, cell adhesion, dendritic morphology, axonal guidance, and synapse formation and regulates neuromuscular synapse organization and target selection [39,43]. Mutations in the human *TEN4* gene are associated with essential tremor movement disorder in patients [44]. Ten4^−/−^ mice exhibit a tremor-like phenotype, and a missense mutation in the *TEN1* gene is related to disorders affecting movement and posture [45].

σ1R is highly expressed in motor neurons [1,27], and autosomal recessive loss-of-function mutations in σ1R are primarily associated with distal hereditary motor neuropathy [10,46,47], and ALS/FTLD [23,48]. In vitro studies have revealed aberrant subcellular distribution of σ1R E102Q in NSC34 cells and have shown that cells expressing the mutant protein are more prone to apoptosis induced by ER stress than those expressing the WT protein [25]. Moreover, expression of the σ1R E102Q mutant protein reduces mitochondrial ATP production, inhibits proteasome activity, and causes mitochondrial injury, aggravating ER stress-induced death of neuro2A cells [49]. Additionally, in cultured hippocampal neurons, overexpression of the σ1R E102Q mutant destabilizes mushroom spines [50]. The σ1R E102Q mutant protein aggregates and accumulates in the ER and associated compartments in transfected cells, provoking alterations in proteasomal degradation and calcium homeostasis [30]. Given the functional relation between HINT1 and σ1R, alterations in the normal functioning of any of these proteins may contribute to the onset of motor neuron pathology.

Various exogenous ligands and neurosteroids alter the calcium-dependent association of σ1R with regulated proteins [11,31,33,36]. Depending on the interacting protein, the same σ1R ligand can either promote the disruption of the complex or prevent the disrupting activities of other ligands [33]. Thus, σ1R ligands exhibit biased activity to regulate subsets of σ1R interactions with third partner proteins, which can be exploited for the development of site-specific drugs with therapeutic significance. σ1R lacks a GPCR structure and transduction regulation; thus, it is considered as a ligand-operated chaperone [51]. While S1RA and progesterone diminished the association of σ1R with the NR1 subunit, these antagonists failed to alter the association of this subunit with σ1R E102Q. S1RA enhanced and PPCC diminished the σ1R–BiP interaction; however, these ligands did not alter the σ1R E102Q–BiP interaction.

E102 forms a hydrogen bond with Y173 in β10 [52] and a pair of hydrogen bonds with the backbone amide nitrogen atoms of V36 and F37, which are part of a structured tether between the transmembrane domain and cytosolic domain. The E102Q mutation abolishes the negatively charged cluster located in the linker region between β2 and β3 (residues 98–106, ASLSEYVLL) and alters hydrogen-bonding properties at the junction between the N-terminal helix and the C-terminal domain adjacent to the ligand-binding pocket of σ1R [29]. This mutation does not impede the binding of ligands to σ1R, as observed in ligand binding assays [52], but modifies the affinity of σ1R for target proteins and its response to cytosolic calcium levels. It also abolishes the effects of ligand binding on the formation of complexes between σ1R and regulated signaling proteins. σ1R E102Q binds poorly to BiP in the ER and may promote the influx of extracellular calcium through calcium channels such as NMDARs and certain types of TRP channels, contributing to the disruption of cellular calcium homeostasis and the onset of juvenile ALS.

## 4. Materials and Methods

### 4.1. Recombinant Protein Expression

The coding region of full-length murine σ1R (AF004927) and its mutated sequence, HINT1 (NM_008248), the C-terminal region of MOR1 (AB047546: residues 286–398), the C0-C1-C2 region of the glutamate NMDAR NR1 subunit (NM_008169: residues 834–938), and the N- and C-terminal regions of TRPA1 (NP_808449; residues 1–721 and 961–1125, respectively), TRPV1 (NP_542437; residues 1–433 and 680–839, respectively), and TRPM8 (NP_599013; residues 1–639) were amplified by RT-PCR using total RNA isolated from the mouse brain as a template.

Specific primers containing an upstream Sgf I restriction site and a downstream Pme I restriction site were used as described previously [31]. The PCR products were cloned downstream of the glutathione S-transferase (GST)/HaloTag coding sequence (Flexi Vector, Promega Biotech Iberica, Madrid, Spain) and the tobacco etch virus (TEV) protease site, and sequencing revealed that the sequences of the proteins were identical to the GenBank sequences. The vectors were introduced into *Escherichia coli* BL21 (KRX #L3002, Promega), and clones were selected on solid medium containing ampicillin. After 3 h of induction at room temperature (RT) in the presence of 1 mM isopropyl β-D-1-thiogalactopyranoside (IPTG) and 0.1% rhamnose, cells were collected by centrifugation and maintained at −80 °C.

The fusion proteins were purified under native conditions on GStrap FF columns (#17-5130-01, GE Healthcare, Madrid, Spain) or with HaloLink resin (#G1915, Promega). When necessary, retained fusion proteins were cleaved on the column with ProTEV protease (#V605A, Promega), and further purification was achieved by high-resolution ion-exchange separation (#780-0001Enrich Q, BioRad Laboratories, Madrid, Spain). Sequences were confirmed by automated capillary sequencing. Recombinant calmodulin (CaM, #208694, Merck-Millipore Iberica, Madrid, Spain) and BiP (#ab78432, Abcam, Cambridge, UK) were obtained from commercial sources.

### 4.2. In Vitro Interactions between Recombinant Proteins: Pull-Down of Recombinant Proteins

Recombinant WT σ1R and σ1R E102Q (100 nM) were incubated with either Sepharose® 4B (#17-0120-01, GE Healthcare; negative control) or with immobilized proteins, including the C-terminus of MOR1, the C0-C1-C2 region of the NMDAR NR1 subunit, the N- and C-terminal domains of TRP, HINT1, and BiP, which were covalently attached to NHS-activated Sepharose® 4 Fast Flow (4FF, #17-0906-01, GE Healthcare) according to the manufacturer’s instructions.

The interactions were studied in 300 µL of buffer containing 50 mM Tris-HCl (pH 7.4) and 0.2% 3-[(3-cholamidopropyl)dimethylammonio]-1-propanesulfonate (CHAPS) in the presence of 2.5 mM CaCl_2_ and mixed by rotation for 30 min at RT. After incubation, the pellets were recovered by centrifugation, washed three times with 2.5 mM CaCl_2_, solubilized in 2× Laemmli buffer, and analyzed by western blotting.

The influence of added calcium on the association of WT σ1R and σ1R E102Q with the C0-C1-C2 region of the NR1 subunit, the C-terminus of TRPV1, and BiP was also evaluated. The recombinant σ1R and its mutant (100 nM) were incubated either alone (negative control) or with immobilized proteins in 300 μL of buffer containing 50 mM Tris-HCl (pH 7.4) and 0.2% CHAPS in the presence of increasing amounts of calcium chloride for 30 min at RT. This protocol was also carried out to assess the competition between CaM and high concentrations of WT σ1R and the E102Q mutant for binding to the C0-C1-C2 region of the NR1 subunit.

To evaluate the effect of σ1R ligands on the association between σ1R E102Q and the NR1 subunit and between σ1R E102Q and BiP, agarose-attached protein–σ1R E102Q complexes were incubated for an additional 30 min at RT with rotation in the presence of increasing concentrations of ligand in a final reaction volume of 300 μL of 50 mM buffer containing Tris-HCl (pH 7.4), 2.5 mM CaCl_2_, and 0.2% CHAPS. If an organic solvent was required for incorporation of the studied ligand, such as dimethyl sulfoxide (DMSO), the concentration of the solvent in the assay buffer remained below 1%. Agarose pellets containing bound proteins were obtained as described above. The following compounds were studied: progesterone (#P7556, Sigma-Aldrich Química, Madrid, Spain), pregnenolone sulfate (#P162, Sigma-Aldrich), S1RA (#16279, Cayman Chemical, Ann Arbor, MI, USA), and PPCC (#3870, Tocris Bioscience, Bristol, UK).

### 4.3. Western Blotting

Unbound WT σ1R and σ1R E102Q proteins recovered by the procedure described above were resolved by SDS-PAGE on 4–12% Bis-Tris gels (#NP0341, Invitrogen, Fisher Scientific) with MES SDS running buffer (#NP0002, Invitrogen). The proteins were transferred onto 0.2 μm polyvinylidene difluoride (PVDF) membranes (#162-0176, BioRad) and probed overnight at 6 °C with anti-σ1R (#42-3300, Invitrogen, Fisher Scientific, Madrid, Spain) and anti-CaM (#05-173, Merck-Millipore) primary antibodies diluted in Tris-buffered saline (pH 7.7) (TBS) + 0.05% Tween 20 (TTBS). All primary antibodies were detected using appropriate horseradish peroxidase-conjugated secondary antibodies, which were visualized by chemiluminescence (#170-5061, BioRad) and imaged on an ImageQuant LAS 500 system (GE Healthcare). Single bands of the expected size were observed because all the assays were performed with recombinant proteins, and these bands were used for subsequent densitometric analysis. Accordingly, there were no bands on other areas of the blots, and these areas were routinely excluded from the analysis. The software automatically calculated the optimal exposure time for obtaining the strongest possible signal for each blot, allowing the bands to be accurately quantified. For each group of samples, the area of the strongest protein immunosignal was measured (average optical density of the pixels within the object area/mm^2^; AlphaEase FC software, Alpha Innotech, San Leandro, CA, USA). The gray values of the means were then normalized within the 8 bit/256 gray levels (256-computed value/computed value).

### 4.4. Statistical Analyses

The western blot data were expressed as the change in signal relative to that of the control group, which was assigned an arbitrary value of 1. Statistical analyses were performed using the Sigmaplot/SigmaStat v. 14 package (Statistical Package for The Social Sciences (SPSS) software, Erkrath, Germany), and *p* < 0.05 was considered to indicate significance. The data were analyzed using one-way ANOVA followed by the pairwise Holm–Sidak multiple comparison test.

## Supplementary Materials

Supplementary materials can be found at https://www.mdpi.com/1422-0067/21/19/7339/s1. Figure S1. In vitro assay of the binding of σ1R to different signaling proteins.

## Abbreviations

ALS	Amyotrophic lateral sclerosis
BiP	Binding immunoglobulin protein
CaM	Calmodulin
CHAPS	3-[(3-cholamidopropyl)dimethylammonio]-1-propanesulfonate
ER	Endoplasmic reticulum
FTLD	Frontotemporal lobar degeneration
GPCR	G-protein coupled receptor
HINT1	Histidine triad nucleotide-binding protein 1
IP3R3	Inositol 1,4,5-triphosphate receptor type 3
MAM	Mitochondria-associated ER membranes
MOR	Mu-opioid receptor
NMDAR	*N*-methyl-D-aspartate receptor
PPCC	2-[(4-Hydroxy-4-phenyl-1-piperidinyl)methyl]-1-(4-methylphenyl)-cyclopropanecarboxylic acid
S1RA	4-[2-[[5-methyl-1-(2-naphthalenyl)-1H-pyrazol-3-yl]oxy]ethyl] morpholine
σ1R	Sigma 1 receptor
TRP	Transient receptor potential calcium channel
WT	Wild-type
