# Supplementary material for: The ALS-Related σ1R E102Q Mutant Eludes Ligand Control and Exhibits Anomalous Response to Calcium
Source: Int J Mol Sci. 2020 Oct 4;21(19):7339. doi: 10.3390/ijms21197339 (PMC7582951; doi:10.3390/ijms21197339)
Supplement: Supplementary file 1 [file ijms-21-07339-s001.pdf]

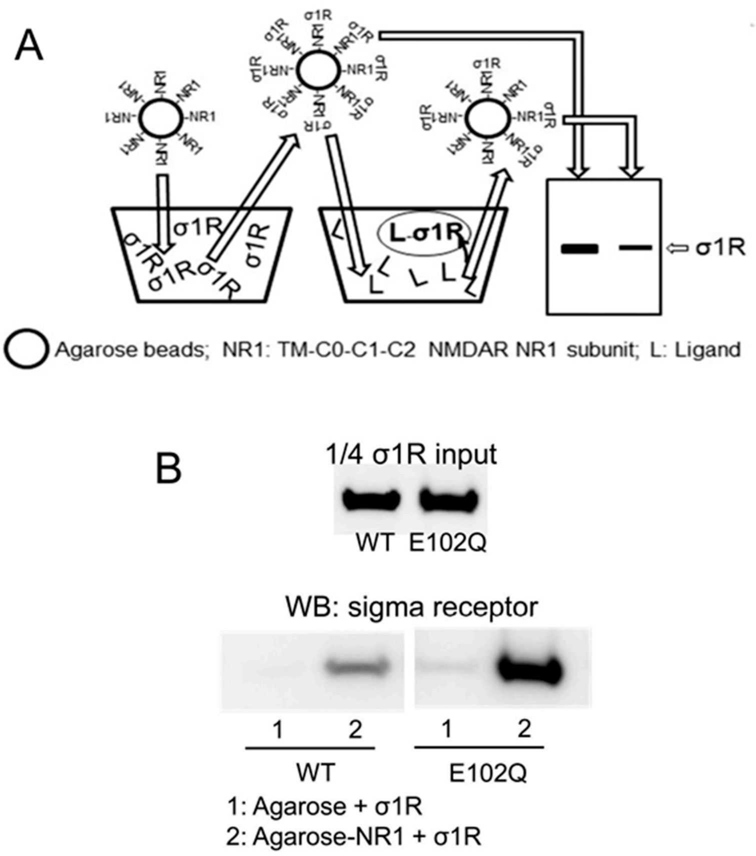

Figure 1. In vitro assay of the binding of σ1R to different signaling proteins. (**A**) NHS-activated agarose (Sepharose®) beads were covalently coupled to the N-terminus of the studied protein (NR1 subunit of glutamate NMDA receptor is shown here) and incubated with excess σ1R (at a concentration determined to induce maximum binding to the agarose-coupled protein by a pilot assay). The unbound σ1R was washed out, and depending on the type of study, protein-bound σ1R was analyzed immediately or exposed to serial concentrations of the studied ligands. σ1R that remained attached to the NR1 subunit was then evaluated by SDS-PAGE and immunoblotting (see the Methods section). (**B**) Recombinant WT σ1R and E102Q mutant bound to NHS-activated agarose–C0-C1-C2 region of the NR1 subunit, but not to inactivated NHS-Sepharose® (negative control). Recombinant cytosolic C0-C1-C2 region of the NMDAR NR1 subunit was covalently attached to NHS-activated Sepharose® and incubated with WT σ1R or mutant σ1R (100 nM) in the presence of 2.5 mM $CaCl_2$. NHS, N-Hydroxysuccinimidyl TM, transmembrane; C0-C1-C2, cytosolic region of NR1 subunit of the NMDA receptor; WB, western blot; WT, wild-type.
